# A New Gorilla Adenoviral Vector with Natural Lung Tropism Avoids Liver Toxicity and Is Amenable to Capsid Engineering and Vector Retargeting

**DOI:** 10.1128/JVI.00265-20

**Published:** 2020-05-04

**Authors:** Zhi Hong Lu, Igor P. Dmitriev, Douglas E. Brough, Elena A. Kashentseva, Jie Li, David T. Curiel

**Affiliations:** a Department of Radiation Oncology, Biologic Therapeutics Center, Washington University School of Medicine, St. Louis, Missouri, USA; b Precigen, Inc., Germantown, Maryland, USA; University of California, Irvine

**Keywords:** Gorilla, adenovirus, gene therapy, lung-targeting vector

## Abstract

Human adenoviruses have many attractive features for gene therapy applications. However, the high prevalence of preexisting immunity against these viruses in general populations worldwide has greatly limited their clinical utility. In addition, the most commonly used human adenovirus, human adenovirus subgroup C serotype 5 (HAd5), when systemically administered, triggers systemic inflammation and toxicity, with the liver being the most severely affected organ. Here, we evaluated the utility and safety of a new low-seroprevalence gorilla adenovirus (GAd; GC46) as a gene transfer vector in mice. Biodistribution studies revealed that systemically administered GAd had a selective and robust lung endothelial cell (EC) tropism with minimal vector expression throughout many other organs and tissues. Administration of a high dose of GAd accomplished extensive transgene expression in the lung yet elicited no detectable inflammatory histopathology in this organ. Furthermore, GAd, unlike HAd5, did not exhibit hepatotropism or induce liver inflammatory toxicity in mice, demonstrating the exceptional safety profile of the vector vis-à-vis systemic utility. We further demonstrated that the GAd capsid fiber shared the flexibility of the HAd5 equivalent for permitting genetic modification; GAd with the pan-EC-targeting ligand myeloid cell-binding peptide (MBP) incorporated in the capsid displayed a reduced lung tropism and efficiently retargeted gene expression to vascular beds in other organs.

**IMPORTANCE** In the aggregate, our mouse studies suggest that GAd is a promising gene therapy vector that utilizes lung ECs as a source of therapeutic payload production and a highly desirable toxicity profile. Further genetic engineering of the GAd capsid holds the promise of *in vivo* vector tropism modification and targeting.

## Abstract (precis)

In the aggregate, our mouse studies suggest that GAd is a promising gene therapy vector that utilizes lung ECs as a source of therapeutic payload production and a highly desirable toxicity profile. Further genetic engineering of the GAd capsid holds the promise of *in vivo* vector tropism modification and targeting.

## INTRODUCTION

For a wide range of genetic and acquired diseases, gene-based therapy holds the promise of significant advantages over small-molecule therapeutics. Foremost among these is the increased treatment specificity achieved via targeted therapeutic payload delivery to organ sites and cell types relevant to diseases. In this regard, vectors engineered from adenovirus (Ad) fulfill many desired properties that have promoted their use as a gene therapy delivery platform. These include viral genome stability; large foreign DNA insertion capacity; ease of high-titer viral production; episomal transduction, mitigating the risk of insertional mutagenesis; the ability to infect both dividing and nondividing mammalian cells; and amenability to genetic engineering for vector tropism modification. Consequently, Ad vectors have been one of the most commonly used gene transfer vehicles in gene therapy clinical trials worldwide since their initial approval in 1989 (1).

To date, among the identified Ad serotypes from different species, human adenovirus subgroup C serotype 5 (HAd5) is by far the most extensively studied member for virus biology and genetic engineering and has consequently been utilized in a majority of Ad-based gene therapy clinical trials. However, accumulating clinical data have revealed that these vectors face major limitations in providing efficient gene transfer to target tissues for therapeutic benefits. This inadequacy is partly due to the high prevalence of preexisting immunity against HAd5 in world populations, which can neutralize adenovirus infectivity. In addition, systemically administered HAd5 displays a strong liver tropism and, when high concentrations of the vector are delivered, can trigger severe innate and adaptive immune responses against infected cells, resulting in liver damage. This dose-limiting hepatotoxicity represents a major barrier to high-dose-dependent gene transfer to extrahepatic tissues. For these reasons, there has been great interest in developing alternative vectors based on low-prevalence Ad serotypes from human and various animals, including those of porcine, canine, bovine, ovine, and nonhuman primate origin. Although these serotypes share similar genome structures, they are tremendously diverse in their respective cellular receptors, *in vivo* biodistribution patterns, and innate/adaptive immune activities, thus providing a growing pool of candidates for development of alternative safer therapeutic vectors for individuals with preexisting immunity against HAd5.

Recently, three adenovirus isolates derived from wild gorillas (gorilla adenovirus [GAd] type 9, isolates 44, 45, and 46) have been developed as replication-deficient vectors that allow a high-titer yield in standard packaging cell lines (2). These adenoviruses are closely related to the human species C group yet show a very low seroprevalence in general populations (3, 4). Initial preclinical vaccine studies have shown that these vectors are as effective as HAd5 in inducing high-level antigen-specific antibody and T-cell responses from a single or repeated administration (5). Here, we evaluated for the first time the utility of one of the vectors (gorilla adenovirus type 9, isolate 46 [here, GAd]) as a gene transfer vector in mice. Our results revealed that systemically administered GAd had a strong lung endothelial cell (EC) tropism with minimal vector expression throughout host organs, including liver, brain, heart, kidney, muscle, and small and large bowel. While freely circulating GAd particles, like those of HAd5, were cleared predominantly by Kupffer cells in liver, the lack of hepatotropism of the vector was associated with none of the detectable liver inflammation or toxicity that was seen with the same dose of HAd5. Interestingly, GAd, when intravenously administered at a dose producing extensive and robust transgene expression in the lung, elicited only a low inflammatory response and no detectable histopathology in this organ. Here, we also endeavored proof-of-principle studies to demonstrate that GAd capsid fiber, like the HAd5 equivalent, allowed genetic modification. Of note in this regard, GAd incorporating the pan-EC-targeting ligand myeloid cell-binding peptide (MBP) displayed a reduced lung tropism and efficiently retargeted gene expression to vascular beds in other organs, including heart, small intestine, muscle, and brain. In sum, GAd displayed a highly selective lung EC tropism, exceptional host tolerability of a high dose of the vector, and vector targeting via virus capsid modification.

## RESULTS

### GAd exhibited natural lung EC tropism following systemic administration.

**FIG 1:**
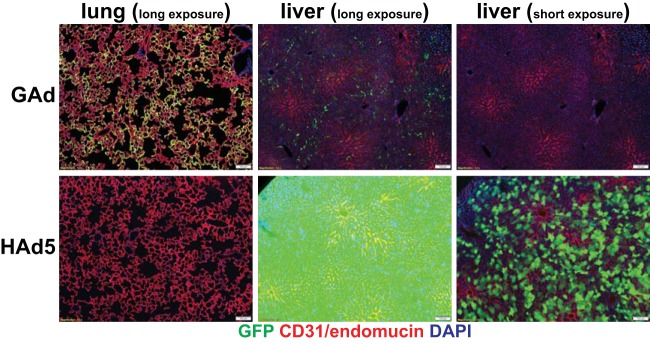
GAd showed a strong lung tropism and minimal liver cell transduction. Immunofluorescence microscopy was used to analyze vector GFP expression in lung and liver from mice at day 3 following intravenous injection of 1.0 × 10^11^ viral particles (vp) of GAd (*n* = 9) and HAd5 (*n* = 9). The tissue sections were counterstained with vascular EC markers CD31 and endomucin. Magnifications, ×100. Red, CD31/endomucin; green, GFP immunofluorescence; blue, DAPI. The GFP fluorescence exposure time was 120 ms for the lung long exposure, 120 ms for the liver long exposure, and 15 ms for the liver short exposure.

**FIG 2:**
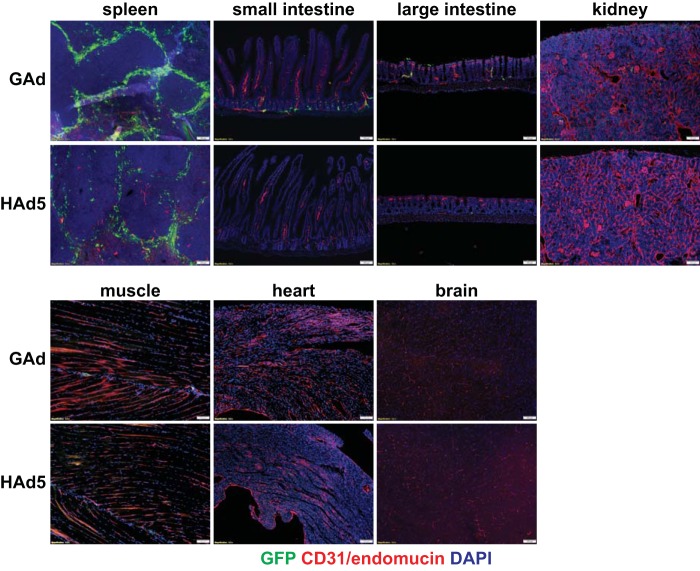
Biodistribution of GAd and HAd5 vector transgene expression in mice. Immunofluorescence microscopy analysis of vector GFP expression in host organs at day 3 following intravenous injection of 1.0 × 10^11^ viral particles (vp) of GAd (*n* = 3) and HAd5 (*n* = 3) into C57BL/6J mice revealed strong splenic marginal zone macrophage transduction and sporadic transgene expression in the small and large bowel, nearly absent expression in kidney and muscle, and no detectable expression in heart and brain. Costaining of tissue sections with an EC-specific CD31/endomucin cocktail revealed that enhanced GFP expression was restricted to the vasculature in nonspleen organs. Magnifications, ×100. Red, CD31/endomucin; green, GFP; blue, DAPI. The GFP fluorescence exposure time was 120 ms for all organs.

**FIG 3:**
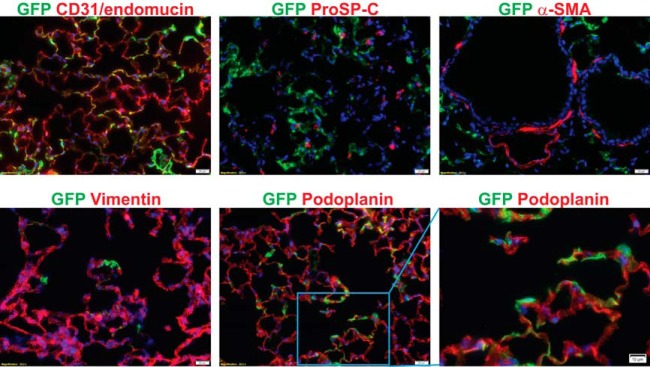
GAd vector expression in lung was endothelial cell restricted. Coimmunofluorescence staining of cellular markers for alveolar type I and type II epithelial cells (podoplanin and ProSP-C, respectively), myofibroblasts (α-SMA), stromal fibroblasts (vimentin), and differentiated hematopoietic cells (CD45) indicated that the locations of these cell types were distinct from those of GFP-expressing cells. Immunofluorescence colocalization was detected between GFP and endothelial markers CD31 and endomucin. Magnifications, ×400. Red, each stromal protein; green, enhanced GFP immunofluorescence; blue, DAPI.

To evaluate the utility and safety of the gorilla Ad (GC46) as a gene transfer vector, a replication-deficient vector was created by replacing the open reading frames of E1A and E1B with an expression cassette consisting of a green fluorescent protein (GFP) or mCherry reporter gene driven by the cytomegalovirus (CMV) promoter. We first compared the body-wide biodistribution of GAd versus that of HAd5 in mice following systemic vector administration using immunofluorescence microscopy analysis. As expected, HAd5 vector gene expression was predominantly in liver hepatocytes (Fig. 1) and readily found in spleen marginal zone macrophages (Fig. 2). In contrast to the liver tropism of HAd5, GAd efficiently transduced cells in the lung, an organ refractory to HAd5 infection. Of note, the GAd vector expression level in liver was drastically lower than GAd lung expression and HAd5 liver expression (Fig. 1). GAd transduced the spleen marginal zone at levels comparable to those of HAd5 (Fig. 2). Elsewhere, small and large intestines showed occasional GAd vector expression, while kidney, skeletal muscle, heart, and brain were devoid of detectable transduction (Fig. 2). We next performed coimmunofluorescence staining of GAd-infected lung sections with multiple pulmonary cell-type markers to identify the GAd-targeted cell type(s). High-power field imaging revealed that GFP reporter immunofluorescence was localized to the endothelial celsl (ECs) of alveolar sac/wall capillaries (Fig. 3, CD31/endomucin positive [endomucin^+^]). The reporter immunofluorescence location was clearly distinct from alveolar type II cells (prosurfactant protein C [ProSP-C] positive), myofibroblasts (alpha smooth muscle actin [α-SMA] positive), fibroblasts (vimentin positive), and differentiated cells of hematopoietic origin (CD45^+^). The GFP immunofluorescence was adjacent to the podoplanin-positive alveolar type I cells, consistent with GAd EC-specific expression in lung. Taken together, these data suggested that GAd possesses a natural lung EC tropism with a liver-untargeting effect.

**FIG 4:**
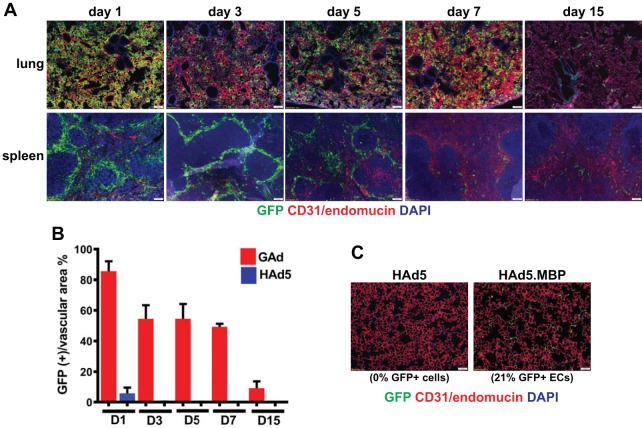
Kinetics of GAd vector expression in lung. (A) Immunofluorescence microscopy analysis of vector GFP expression in lung and spleen from mice at days 1, 3, 5, 7, and 15 following intravenous injection of 1.0 × 10^11^ viral particles (vp) of GAd (2 mice for each time point) revealed that GAd induced rapid, efficient, and transient transgene expression in both organs. Magnifications, ×100. Red, CD31/endomucin; green, GFP; blue, DAPI. (B) The percentage of vascular EC (CD31/endomucin^+^) area expressing GFP in lung from GAd- and HAd5-injected mice at days 1, 3, 5, 7, and 15 (2 mice for each virus and time point) was measured. The bar graph shows the means and standard deviations of the measurements. D, day. (C) Immunofluorescence microscopy analysis of vector GFP expression in lung at day 3 following intravenous injection of 1.0 × 10^11^ viral particles (vp) of HAd5 (*n* = 5) and HAd5.MBP (*n* = 5) into adult C57BL/6J mice revealed that HAd5.MBP was less efficient in lung EC gene transfer than GAd. The percentage of the vascular EC area expressing GFP is reported under each micrograph. Magnifications, ×100. Red, CD31/endomucin; green, GFP; blue, DAPI.

We next further characterized GAd-mediated transgene expression in the lung and spleen. The GFP reporter fluorescence in lung rose rapidly after virus injection, with the level reaching a peak by day 1 and remaining high on days 3, 5, and 7 (Fig. 4A, lung). Quantification of the percentage of lung EC area expressing GFP (GFP^+^) revealed that GAd mediated gene transfer to 83% of lung capillary ECs on day 1, and GFP^+^ ECs were maintained at approximately 50% from day 3 to day 7 and at 9% by day 15 (Fig. 4B). Of note, the lung EC gene transfer efficiency by GAd (52% GFP^+^) at day 3 was at least 2.4-fold of what was attained by the EC-targeted Ad5 with myeloid cell-binding peptide (MBP)-incorporated capsid fiber (HAd5.MBP) in a side-by-side comparison experiment (Fig. 4C, HAd5.MBP; 21% GFP^+^ ECs) (6). GAd exhibited a shorter duration of vector expression in spleen for up to 5 days post-virus injection, and the expression was reduced by day 7 (Fig. 4A, spleen). Thus, these transgene expression kinetics data suggest that GAd conferred a rapid onset of robust gene expression in the lung.

### GAd mitigated liver toxicity and elicited a low inflammatory response in lung.

**FIG 5:**
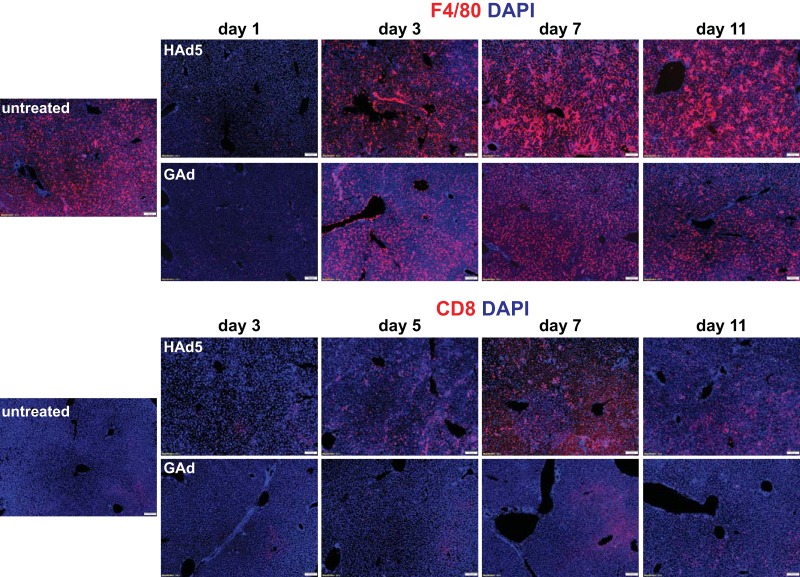
Rapid kinetics of liver Kupffer cell depletion followed by low levels of inflammatory cell infiltration to liver in mice following intravenous injection of GAd. Immunofluorescence microscopy analysis of F4/80^+^ macrophages (top two rows) and CD8^+^ T cells (bottom two rows) in liver from untreated C57BL/6J mice (untreated, *n* = 3) and from mice at days 1, 3, 5, 7, and 11 following intravenous injection of 1.0 × 10^11^ viral particles (vp) of HAd5 and GAd (*n* = 2 for each virus and time point). Magnifications, ×100. Red, CD31/endomucin; green, GFP; blue, DAPI.

We next sought to evaluate whether the low liver gene transfer efficiency of GAd was correlated with reduced liver inflammatory damage compared with that caused by HAd5. As expected, the intravenously injected HAd5 particles were predominantly taken up by phagocytic F4/80^+^ Kupffer cells in liver (data not shown), and the viral uptake triggered depletion of the Kupffer cells by day 1 post-virus administration (Fig. 5, F4/80, HAd5, day 1). Intravenously injected GAd also caused the nearly complete depletion of liver Kupffer cells by day 1, revealing the similar cellular mechanism responsible for the clearance of circulating GAd (Fig. 5, F4/80, GAd, day 1). Consistent with the notion that both innate immunity and adaptive immunity contribute to HAd5-induced liver toxicity (7, 8), mice injected with HAd5 exhibited an increased infiltration of F4/80^+^ macrophages and CD8^+^ cytotoxic lymphocytes into the liver. The infiltration kinetics were evident by day 3 and persisted through day 11 (Fig. 5, HAd5, F4/80 and CD8). In contrast, mice injected with GAd restored the liver F4/80^+^ cell population within the non-virus-injected normal ranges and displayed a greatly attenuated number of liver CD8^+^ infiltrates from day 3 to day 11 (Fig. 5, GAd, F4/80 and CD8).

**FIG 6:**
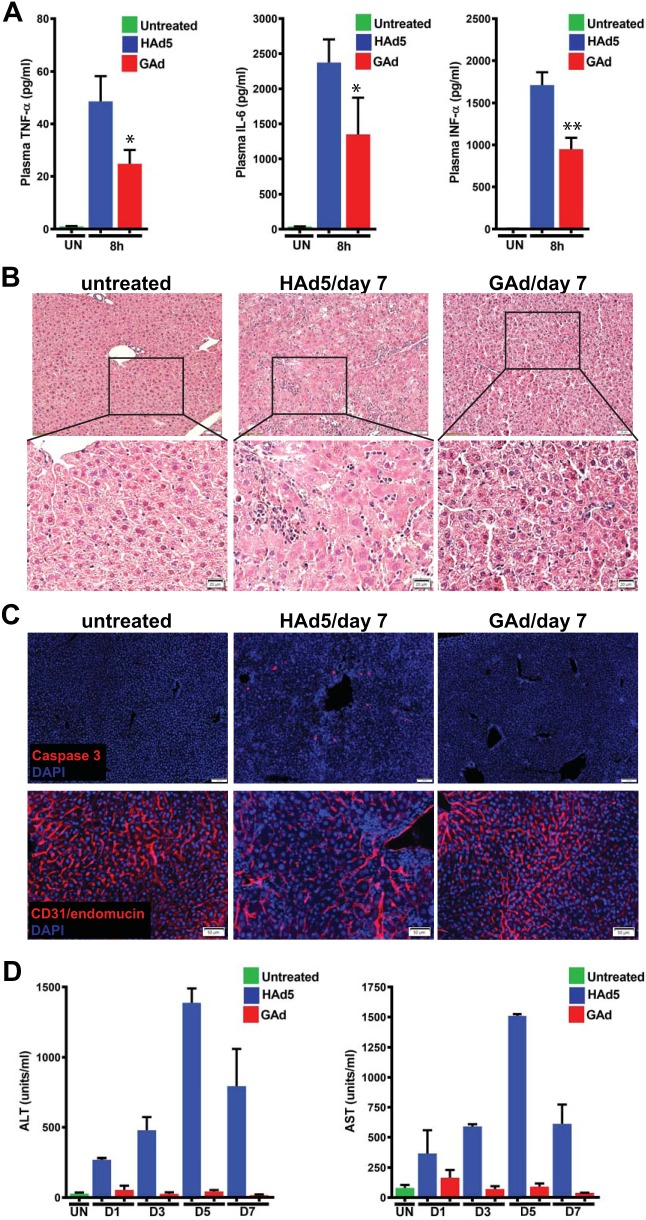
Intravenously injected GAd did not elicit inflammatory toxicity in liver. (A) At 8 h following intravenous injection of 1 × 10^11^ viral particles (vp) of HAd5 and GAd, plasma samples were collected and the levels of TNF-α, IL-6, and IFN-α were measured and compared with those in untreated littermate mice (UN). The bar graphs show the means and standard deviations of the measurements (*n* = 3 for untreated mice and both virus-infected groups). *P* values were determined by unpaired Student *t* tests. *, *P* < 0.05; **, *P* < 0.01. (B) (Top) The same liver samples shown in Fig. 5 were subjected to histopathological analysis with hematoxylin and eosin (H&E) staining. Magnifications, ×100. (Bottom) Enlarged images of the area in squares in the micrographs in the top row. The enlarged image of the HAd5/day 7 micrograph showed increased space between the dystrophic hepatocytes with an enlarged clarified cytoplasm and infiltration of leukocytes around blood vessels. (C) Immunofluorescence microscopy analysis of cleaved caspase 3-positive (top) and CD31/endomucin^+^ cells (bottom) in liver from untreated adult C57BL/6J mice (*n* = 3) and from mice at day 7 following intravenous injection of 1.0 × 10^11^ viral particles (vp) of HAd5 (*n* = 2) and GAd (*n* = 2). (D) At days 1, 3, 5, and 7 following intravenous injection of 1 × 10^11^ viral particles (vp) of HAd5 and GAd, sera were collected and the levels of alanine transaminase (ALT) and aspartate transaminase (AST) were measured and compared with those in untreated littermate mice. The bar graphs show the means and standard deviations of the measurements (*n* = 3 for the untreated group and *n* = 2 for each virus and time point).

Of note, following the sequestration of HAd5, activated Kupffer cells and splenic macrophages are the major source of acutely increased systemic levels of proinflammatory cytokines, which, in turn, contribute to acute inflammatory responses in systemic organs, including liver. Interestingly, while intravenously injected GAd and HAd5 exhibited similar Kupffer cell clearance and splenic marginal zone macrophage infectivity, GAd induced significantly lower plasma levels of proinflammatory cytokines, including tumor necrosis factor alpha (TNF-α), interleukin-6 (IL-6), and alpha interferon (IFN-α), than HAd5 did (Fig. 6A). Next, histological and immune-histological examination of liver sections confirmed the HAd5-induced liver injury, evidenced by markedly reduced hepatic cellularity and leukocyte-populated hepatic lobules (Fig. 6B), an increased hepatic apoptotic index (Fig. 6C, Caspase 3), and markedly dilated blood vessels (Fig. 6C, CD31/endomucin) by day 7 post-virus administration but revealed no detectable hepatic damage from GAd-injected mice by the same time (Fig. 6B and C, GAd/day 7). We next performed liver function tests based on the measurement of the serum levels of alanine transaminase (ALT) and aspartate transaminase (AST). ALT and AST are specifically expressed by hepatocytes but released from damaged cells into the bloodstream; increased serum ALT and AST levels thus can serve as a marker for vector-induced liver damage. Consistent with the kinetics of inflammatory cell infiltration and histomorphologic abnormality in HAd5-infected liver, serum ALT and AST levels rose steadily from day 1, peaked by day 5, and were resolving by day 7 (Fig. 6D, HAd5). In contrast, serum ALT and AST levels in GAd-injected mice remained within the non-virus-injected control range from day 1 to day 7 post-virus administration (Fig. 6D, GAd). As such, these data suggest that GAd, unlike HAd5, did not elicit adverse inflammatory toxicity in liver.

**FIG 7:**
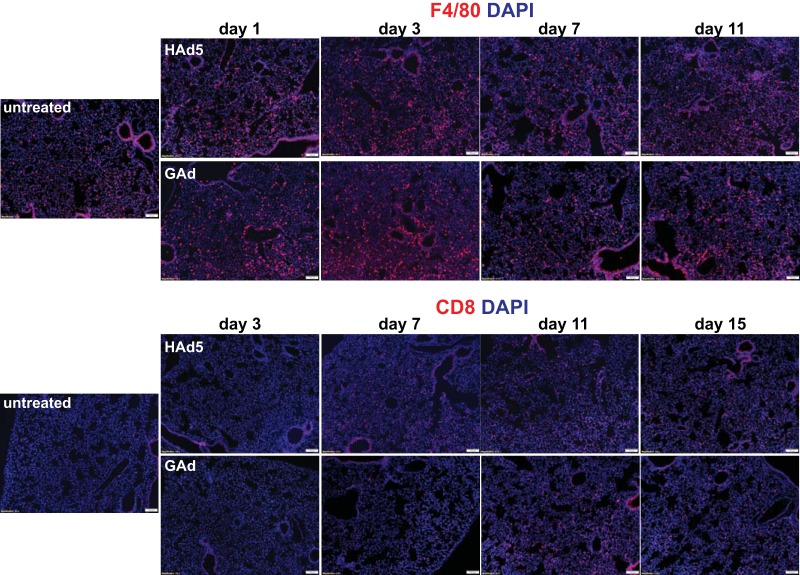
Lack of substantial inflammatory cell infiltration into lung in mice following intravenous injection of GAd. Immunofluorescence microscopy was used to analyze F4/80^+^ macrophages and CD8^+^ T cells in lungs from untreated C57BL/6J mice (untreated, *n* = 3) and from mice at days 1, 3, 7, and 11 following intravenous injection of 1.0 × 10^11^ viral particles (vp) of HAd5 and GAd (*n* = 2 for each virus and time point). Magnifications, ×100. Red, CD31/endomucin; green, GFP; blue, DAPI.

**FIG 8:**
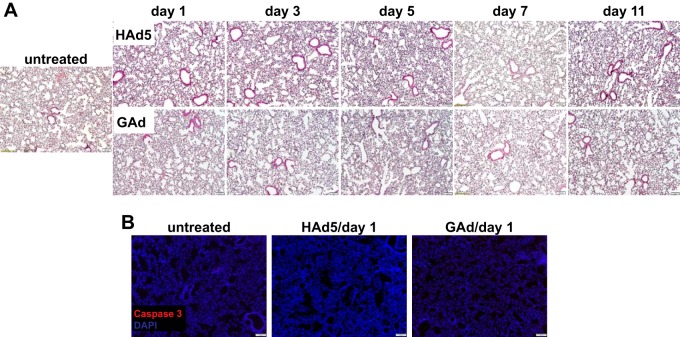
Intravenously injected GAd did not elicit lung damage. (A) Histological analysis of lungs from untreated adult C57BL/6J mice (*n* = 3) and from mice at days 1, 3, 5, 7, and 11 following intravenous injection of 1.0 × 10^11^ viral particles (vp) of HAd5 (*n* = 2) and GAd (*n* = 2). (B) The same lung samples were subjected to immunofluorescence microscope analysis for cleaved caspase 3-positive cell detection. Magnifications, ×100. Red, caspase 3; blue, DAPI.

We next investigated whether systemically injected GAd elicited inflammatory responses in lung, where GAd had a natural tropism. Interestingly, both GAd and HAd5 produced a comparable small increase in F4/80^+^ and CD8^+^ cell infiltration into the lung by day 3, which was resolved by day 7 (Fig. 7, F4/80 and CD8). Importantly, histological and immunohistological examination revealed that the lung morphology (Fig. 8A) or the apoptotic index (Fig. 8B) from the GAd- and HAd5-injected mice from day 1 to day 11 was not distinguishable from that for the non-virus-injected controls. Thus, our data suggest that the high transduction efficiency of GAd did not lead to undesired inflammatory reactions in the lung.

### GAd with the MBP-incorporated chimeric capsid fiber-T4 fibritin had reduced lung tropism and efficiently retargeted gene transfer to vascular beds in many other organs.

**FIG 9:**
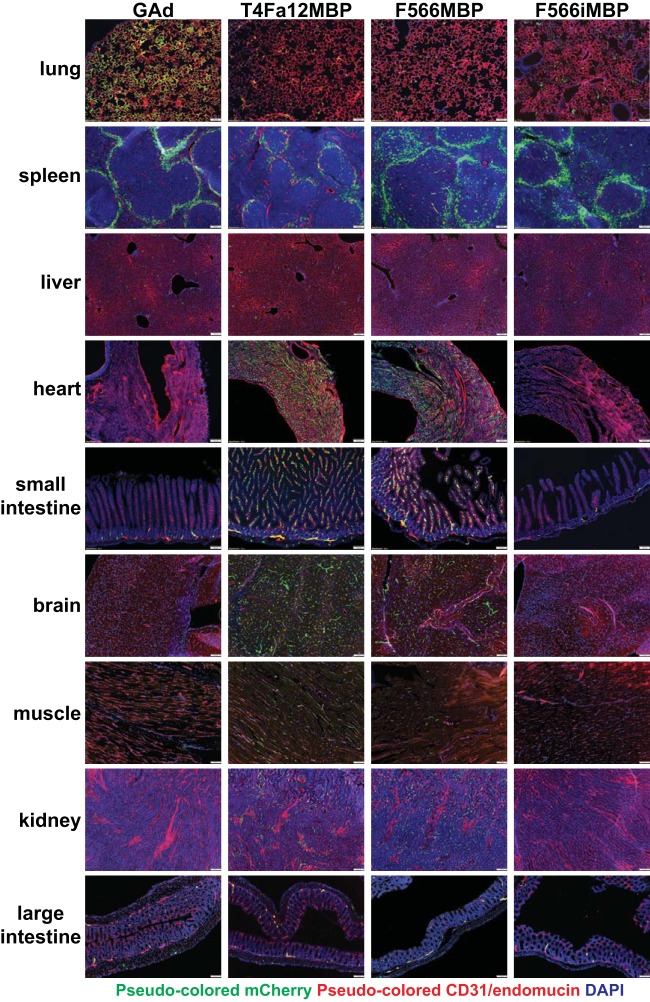
Replacement of GAd fiber knob (knobless) with the T4 fibritin domain tethered with the MBP targeting motif caused reduced GAd.MBP vector expression in lung and retargeting of expression to vascular beds in heart. Immunofluorescence microscopy was used to analyze mCherry reporter expression in lung and heart from C57BL/6J mice at day 3 following intravenous injection of 6.0 × 10^10^ viral particles (vp) of GAd.CMV-mCherry (GAd; *n* = 5), GAd.T4Fa12-MBP.CMV-mCherry (T4Fa12MBP; *n* = 4), GAd.F566-MBP.CMV-mCherry (F566MBP; *n* = 4), and GAd.F566-iMBP.CMV-mCherry (F566iMBP; *n* = 4). Magnifications, ×100. Green, pseudocolored mCherry immunofluorescence signals for consistency of the report of vector expression with that in the rest of work; pseudocolored red, CD31/endomucin; blue, DAPI.

Our group previously showed that capsid-modified HAd5 with the fiber knob deleted and replaced by the MBP ligand-T4 fibritin trimerization domain escaped liver sequestration and enabled multiorgan vascular EC gene transfer (6, 9, 10). In a proof-of-principle experiment to demonstrate the tolerability of GAd to a similar capsid modification, we generated two independent GAd vectors in which the GAd fiber knob was replaced with MBP ligand-T4 fibritin (GAd.MBP) in a slightly different configuration (T4Fa12-MBP [GAd.T4Fa12-MBP] and F566-MBP [GAd.F566-MBP]). Compared with the unmodified GAd, both GAd.MBP vectors produced markedly reduced levels of lung gene transfer and efficiently retargeted gene transfer to vascular ECs in heart, small intestine, brain, skeletal muscle, and kidney microvascular endothelial expression following systemic administration (Fig. 9, F566MBP and T4Fa12MBP). Interestingly, neither the GAd.F566-MBP vector nor the GAd.T4Fa12-MBP vector changed expression in spleen or liver. Importantly, capsid-modified GAd with the nontargeting inverted MBP sequence peptide (iMBP) (GAd.iMBP) incorporated into the GAd fiber-T4 fibritin fusion did not exhibit the heart-, small intestine-, brain-, skeletal muscle-, or kidney-retargeting effect, confirming a crucial role of the MBP ligand in mediating the vascular EC targeting in these organs (Fig. 9, F566iMBP). The GAd.F566-iMBP vector, like the GAd.MBP vectors, exhibited a noticeable reduction of lung EC expression compared with unmodified GAd, implying that the GAd fiber knob removed in the GAd.MBP and GAd.iMBP vectors is an important determinant of GAd lung EC tropism. In sum, these results suggest that GAd, like HAd5, is amenable to capsid engineering and that incorporation of a tissue-specific targeting ligand into the viral capsid can accomplish vector tropism alteration and gene transfer retargeting.

## DISCUSSION

The GAd studied here was originally isolated from a wild gorilla and exhibited a very low seroprevalence in human populations, with the few positive titers also being too low to be inhibitory to the virus (2). The subsequent successful vectorization of the virus embodies significant progress in the development of new gene delivery platforms that are capable of avoiding the problems associated with preexisting immunity against current clinical viral vectors (2, 4, 5). One of the salient features of the GAd-based vector revealed from the present study is its strong native tropism for pulmonary vascular ECs following systemic administration of the vector. For all practical purposes, this E1-deleted unmodified vector is serendipitously a potent lung-targeted gene delivery vehicle with minimal expression in off-target organs. In terms of specificity and the efficiency of pulmonary gene delivery, the GAd vector is superior to the lung-preferred HAd5.MBP vector previously developed by our group (9–11) (data not shown). In fact, the GAd vector may have been the best-performing pulmonary gene delivery platform among various viral and nonviral systems described in the existing literature that the authors are aware of (12, 13). While the precise molecular determinant of the virus tropism specificity remains to be elucidated, our viral capsid modification data suggested an involvement of the fiber knob in modulating the vector lung-targeting efficiency. For intravenously injected GAd, lung is the first-pass organ, and our data are consistent with the notion that the pulmonary vascular ECs with a high vector receptor abundance can sequester the viral particles in a saturable fashion before an excess dose bypassing the lung EC capacity can reach other organs (6, 9).

Gene therapy has long been a sought-after strategy for a range of acquired and inherited diseases of the lung, yet it remains a major challenge for this approach to deliver a sufficient therapeutic payload to the organ to result in the reversal or amelioration of disease symptoms (12, 13). Interestingly, a recent mouse vaccine study showed that a GAd-based immunization vector induced potent and durable immune protection against respiratory infectious disease caused by respiratory syncytial virus (RSV) infection (5). In addition to the genetic vaccine approach for long-term protection of the lung, we also validated the utility of a systemically delivered GAd vector expressing influenza A virus-neutralizing antibodies in providing rapid protection of mice against intranasal influenza virus challenges (unpublished data). As such, the superiority of the GAd vector for the local delivery of genetic therapeutics warrants its potential application in the treatment of a number of lung diseases, such as infections, allergic asthma, and lung injury repair (14–16). Furthermore, the potent vector expression in lung ECs also holds the promise of making lung an important source of therapeutic secretory factors that are released into the circulation for the treatment of systemic diseases (13).

The GAd-mediated GFP reporter gene expression in lung lasted for at least 7 days (Fig. 3). It is interesting to note that an *in vivo* transient expression pattern was seen with E1-deleted HAd5, exhibiting a rapid onset of gene expression in hepatocytes with sustained expression for a few days but an undetectable level of expression after a few weeks (17). It has been well documented in the literature that the major cause of HAd5 transgene silencing was the destruction of HAd5-infected cells by cytotoxic T lymphocytes (CTLs) triggered by the immunogenic viral proteins within the infected cells (18). Of note, the second-generation gutless HAd5 vector with the whole viral genome sequences replaced by transgene and stuff DNA along with the two inverted terminal repeats (ITRs) and Ψ packaging element elicited a negligible CTL response and produced a much longer duration of transgene expression (19–21). Although it remains to be established at what level the innate and adaptive immune responses against viral proteins expressed from the GAd genome will occur, creation of an equivalent gutless GAd vector seems to be a testable strategy for future advancement of pulmonary gene therapy. In genetic lung diseases, such as alpha-1 antitrypsin (A1AT) deficiency and pulmonary arterial hypertension, and in systemic diseases like hemophilia A and B, lifelong expression of a corrective gene is a prerequisite for the success of gene therapy. Recently, our group has achieved long-term and possibly permanent corrective gene expression using HAd5 delivery of CRISPR/Cas9 genome-editing machinery. This strategy relied on targeted integration of a therapeutic gene into a genomic safe harbor, the *ROSA26* locus, in mouse hepatocytes (22, 23). Importantly, lineage tracing of pulmonary vascular ECs in mice (24) suggested that the *ROSA26* locus also supported the efficient and stable expression from a knocked-in fluorescent reporter gene without epigenetic silencing in these cells (our unpublished data). Therefore, with the confirmed favorable cellular milieu and chromatin state in the target cells, the next step will be to explore the feasibility of long-term/permanent pulmonary gene therapy using a GAd vector-delivered genome-editing strategy.

A high dose of the lung-tropic E1-deleted GAd following systemic administration triggered minor, controllable innate and adaptive immune responses and did not produce detectable histopathology in the lung (Fig. 5). Nor did the GAd elicit adverse inflammatory responses in liver, while HAd5 at a matching dose caused marked hepatic inflammatory toxicity (Fig. 4). Interestingly, we demonstrated in this study that circulating GAd particles, like their HAd5 counterparts, were subject to sequestration by liver Kupffer cells, indicating an interaction of GAd with the host natural antibodies and complement system that facilitate Kupffer cell uptake of the vector (25). The intravenously delivered viral particles initially bypassing the lung sequestration were at least in part taken up and destroyed by the liver Kupffer cells, leading to massive Kupffer cell depletion in liver at day 1 following virus administration (Fig. 5). In addition to Kupffer cells, spleen marginal zone macrophages are also implicated in harboring HAd5 particles from circulation (26, 27). In this regard, splenic macrophages also sequestered GAd particles efficiently at levels comparable to those for HAd5 (data not shown), yet the level of induction of plasma proinflammatory cytokines, including tumor necrosis factor alpha, interleukin-6, and alpha interferon, following intravenous GAd administration was significantly lower than that following HAd5 administration (Fig. 6A), therefore implying that GAd, unlike HAd5, may have a markedly reduced intrinsic capacity to activate macrophages. Since relatively high doses of GAd were used in this study and with the discovery of splenic macrophage sequestration of the GAd vector, it is critical next to fully understand the *in vivo* vector transduction efficiency in response to a deescalating dose of the vector for an optimized ratio of lung versus spleen gene delivery (28, 29). In terms of gene therapy, HAd5-mediated liver toxicity was the major barrier preventing HAd5 from achieving efficient liver-targeted gene expression at high vector doses. In contrast to the dismal outcomes achieved with the HAd5-based gene therapy vectors, the potential of the unmodified GAd to be an efficient lung-targeted gene therapy vehicle is strengthened by its excellent safety profile in mice.

HAd5 has long been studied for genetic modification of its capsid proteins to achieve an ablation of native tropism and a gain of cell-specific retargeting of vector expression (30). To this end, we confirmed here in a proof-of-principle experiment that the GAd capsid fiber protein is also amendable for genetic engineering, which led to *in vivo* vector tropism modification and vector retargeting (Fig. 6). In sum, GAd embodies an excellent new gene therapy platform that shows a very low seroprevalence of neutralizing immunity in the human population, that exhibits exceptional host tolerability of a high dose of the vector when administered systemically, and that has the potential of broadened tissue- and cell type-specific gene therapy applications through virus capsid modification for tropism alteration and gene transfer retargeting.

## MATERIALS AND METHODS

### Construction of recombinant GAd vectors.

We employed the pACgc46E1(d2t.L) plasmid carrying the GAd GC46 genome containing the firefly luciferase gene driven by the CMV tetO promoter in place of the deleted early E1 region to replace the fiber knob-coding sequence with the *bla* ampicillin resistance gene flanked by an AbsI restriction site on each side. To this end, the DNA fragment including the last 1,029 bp of the fiber gene and 1,020 bp of the downstream E4 region was PCR amplified with Fusion DNA polymerase using primers GAdKn.Fwd (5′-aaa cga cgg cca gtg aat tcG CAA AAC TGG TCG CAC CCC TAG-3′) and GAdKn.Rev (5′-tga cca tga tta cgc cCC AGC TCT ATG GTT CAC GGC TAC-3′), designed using the NEBuilder online tool (uppercase nucleotides are gene specific, and lowercase nucleotides overlap), and then cloned the amplicon into the pUC57kan plasmid between EcoRI and HindIII sites by isothermal DNA assembly with a NEBuilder HiFi DNA assembly cloning kit, as recommended by the manufacturer (New England BioLabs Inc.) The constructed pUC57kGAdknob plasmid was digested with HindIII to replace the last 603 nucleotides (nt) of the fiber gene coding sequence following the last fiber shaft pseudorepeat and knob domain along with the downstream untranslated 90 nt located between HindIII sites with the *bla* ampicillin resistance gene, which was amplified using Fusion DNA polymerase with primers AmpR.Fwd (5′-act agg ctc tgg act aag ctt cct cga ggC ACG TTA AGG GAT TTT GGT CAT G-3′) and AmpR.Rev (5′-tct cat gca gtt tga tta agc ttc ctc gag gGT GGC ACT TTT CGG GGA A-3′) and pUC18 plasmid DNA as the template. The resultant pUC57kGC46dKn-AmpR plasmid was digested with EcoRI and PstI, and a 2,424-bp DNA fragment was employed for homologous recombination with the pACgc46E1(d2t.L) genomic plasmid in Escherichia coli strain BJ5183 as described elsewhere (31). The recombinant plasmid clones selected on ampicillin plates were confirmed to be high-molecular-weight DNAs by agarose gel electrophoresis and then were transformed into E. coli strain DH10B to purify the pGAd(Kn-AmpR)CMV-luc plasmid containing the *bla* gene flanked by AbsI restriction sites in place of the deleted fiber knob domain. This plasmid was digested with SnaBI and used for homologous recombination with a PmeI-linearized pGC46mCherry shuttle plasmid to incorporate the mCherry fluorescent protein reporter gene under the control of the CMV promoter in place of the deleted E1 genes, as described elsewhere (2). The resultant pGAdmCherry-dKnob genomic plasmid was digested with the AbsI restriction enzyme and employed for isothermal DNA assembly with double-stranded DNA fragments encoding a heterologous trimerization domain construct derived from bacteriophage T4 protein fibritin (32), followed by a flexible (Gly4Ser)_3_ linker and myeloid cell-binding peptide (MBP), HSCWTLDRGYCSAE, described previously (9, 33). There were two trimerization domain constructs, designed to include (i) the final 95 residues of the wild-type fibritin (from Ile393 to Ala487), comprising the last two alpha-helical coiled-coil segments and the C-terminal foldon, which was designated F566 and which has been described elsewhere (34), and (ii) the last coiled-coil segment and the C-terminal foldon (residues 418 to 487), designated T4Fa12. Thus, we generated pGAdF566MBP and pGAdT4Fa12MBP GC46-based genomes encoding chimeric fiber-fibritin proteins containing the F566 or T4Fa12 trimerization domain, respectively, followed by a flexible linker to display the C-terminal MBP. The pGAdF566iMBP and pGAdT4FiMBP plasmids carried GAd genomes encoding either the F566 or T4F trimerization domain, respectively, followed by a flexible linker to display the C-terminal MBP. The pGAdF566iMBP plasmid carrying GAd genomes encoding either the F566 trimerization domain, the (Gly4Ser)_3_ linker, and the inverted amino acid sequence of MBP (HSCYGRDLTWCSAE) was made essentially as described above to generate relevant control vectors. The constructed pGAdF566MBP, pGAdT4Fa12MBP, and pGAdF566iMBP plasmids were linearized with PmeI to liberate the left inverted terminal repeat (ITR) of the recombinant viral genome and then transfected into 293F28 cells to rescue the corresponding replication-incompetent GAd vectors. The 293F28 cell line (34, 35) was derived from 293 cells (36) and constitutively expresses the wild-type Ad5 fiber protein to be incorporated into the viral capsids and *trans*-complement the lack of binding of the knobless fiber-fibritin ligand to the Ad native receptor CAR. This in-house system was previously employed to rescue and upscale Ad5-based vectors with genetically altered receptor-binding specificity, including the Ad5MBP vector (9, 33). The GAd.F566-MBP, GAd.T4Fa12-MBP, and GAd.F566i-MBP vectors, propagated using 293F28 cells, were purified using cesium chloride density gradient ultracentrifugation following the final passage in 293 cells in order to produce viral progeny containing only the chimeric fiber-fibritin-MBP. Purified virus preparations were dialyzed against 10% glycerol in phosphate-buffered saline (PBS), and the number of virus particles (vp) was determined using optical density (260 nm) measurement, as described by Mittereder et al. (37), and were as follows: for GAd.F566-MBP, 3.3 × 10^11^ vp/ml; for GAd.T4Fa12-MBP, 3.2 × 10^11^ vp/ml; and for GAd.F566i-MBP, 5.8 × 10^11^ vp/ml.

### Mouse studies.

The Animal Studies Committee of Washington University in St. Louis approved all experimental procedures. Mice of the C57BL/6J mouse background were obtained from The Jackson Laboratory (Bar Harbor, ME, USA), and mice of 7 to 10 weeks of age were used for experiments.

### Virus injection and host organ harvest.

Mice were injected with 1 × 10^11^ or 6 × 10^10^ particles of virus in 200 μl of saline via the tail vein. At designated times post-vector injection, mice were induced into deep anesthesia with 2.5% 2,2,2-tribromoethanol (Avertin; Sigma-Aldrich, St. Louis, MO), and the thorax was opened. A 24-gauge round ball-tipped stainless oral gavage needle was inserted into the left ventricle, and the mouse was perfused with 30 ml of 10% PBS-buffered formalin. Organs were removed and cut into 1- to 2-mm-thick slices. Organ slices underwent postperfusion fixation in formalin at room temperature for 1 to 2 h. Harvested organ tissues were prepared in dual sets of frozen and paraffin embedded sections. For frozen sections, organ slices were cryopreserved in 30% sucrose in PBS at 4°C overnight, embedded in NEG50 mounting medium (Thermo Fisher Scientific, Waltham, MA), and frozen in a 2-methylbutane-containing glass beaker prechilled in liquid nitrogen. Fixed tissues were also switched to graded alcohol and xylene series and embedded in paraffin.

### H&E and immunofluorescence staining.

For histological analysis, 5-μm-thick paraffin block sections were prepared and subject to standard hematoxylin and eosin (H&E) staining by the Elvie L. Taylor Histology Core Facility at the Washington University School of Medicine. For immunofluorescence, frozen tissues were sectioned at 12 μm in thickness. Frozen-section slides were dried at room temperature for 10 min, washed three times in PBS to remove the NEG50 mounting medium, and incubated with a protein block solution (5% donkey serum and 0.1% Triton X-100 in PBS) at room temperature for 1 h and then at 4°C in protein block containing primary antibodies overnight. The primary antibodies used in this study included rat anti-endomucin (1:1,000; catalog number 14-5851-81; eBioscience), Armenian hamster anti-CD31 (1:1,000; catalog number MAB1398Z; MilliporeSigma), chicken anti-GFP (1:400; catalog number A10262; Thermo Fisher Scientific), rabbit anti-mCherry (1:250; catalog number ab213511; Abcam), hamster anti-podoplanin (1:100; catalog number 8.1.1; Developmental Studies Hybridoma Bank), rabbit anti-ProSP-C (1:1,000; catalog number AB3786; MilliporeSigma), rabbit anti-α-SMA (1:100; catalog number ab5694; Abcam), goat antivimentin (1:20; catalog number sc7557; Santa Cruz Biotechnology), rat anti-CD45 (1:25; catalog number 550539; BD Biosciences), rat anti-F4/80 (1:50; catalog number MA1-91124; Thermo Fisher Scientific), rat anti-CD8 (1:20; catalog number 550281; BD Biosciences), and rabbit anti-activated caspase 3 (1:400; catalog number 9661; Cell Signaling Technology). On day 2, the slides were washed three times in PBS, incubated with corresponding 1:400-diluted Alexa Fluor 488- and Alexa Fluor 594-conjugated secondary antibodies (Jackson ImmunoResearch Laboratories, West Grove, PA), and counterstained with SlowFade Gold antifade mounting reagent with 4′,6-diamidino-2-phenylindole (DAPI; Thermo Fisher Scientific). Bright-field micrographs were acquired using an Olympus BX61 microscope equipped with a DP80 dual-sensor monochrome and color camera (Olympus America, Center Valley, PA) with CellSens Dimension imaging software (Olympus Soft Imaging Solutions).

### Immunofluorescence microscopy-based analysis of viral reporter gene expression.

Immunofluorescence micrographs were collected using a DP80 dual-sensor monochrome and color camera. The extended focal imaging (EFI) function was used in the processing of a stack of acquired images of different *z*-dimensional focal planes at 1-mm intervals to yield high-magnification in-focus micrographs with the CellSens Dimension imaging software (Olympus). The optimized camera acquisition time for GFP and mCherry immunofluorescence was set *a priori* for each organ within individual experiments and between experiments where the collected data were determined to be within the linear range. Immunofluorescence micrographs were subjected to measurement of both the color intensity and the color-positive area using the CellSens Dimension image analysis software. To determine the fluorescence intensity, a threshold defining the background signal intensity for each pixel was set using non-virus-injected control tissues. A region of interest (ROI) was drawn over the tissue area, and the pixels of positively identified particles in the ROI, defined as pixels with a color intensity above the background, were identified. The color intensity values from every pixel of positively identified particles were summed. To evaluate the fraction of the vascular EC area expressing GFP or mCherry, the endothelial marker-positive area and the reporter-positive area within the tissue ROI were quantified, and the GFP-positive (mCherry-positive) area was calculated as a percentage of the EC-positive area in the micrograph.

### Measurement of serum levels of ALT and AST enzyme activities and plasma levels of TNF-α, IL-6, and IFN-α.

Mice were injected intravenously with 1.0 × 10^11^ vp of the HAd5 and GAd vectors, and at designated times after the injection, sera were collected from the animals. The levels of enzymatic activity of alanine aminotransferase (ALT) and aspartate aminotransferase (AST) were assayed with ALT and AST activity assay kits (MilliporeSigma) according to the manufacturer’s protocol. The plasma levels of TNF-α, IL-6, and IFN-α were assayed using R&D Systems Quantikine enzyme-linked immunosorbent assay kits according to the manufacturer’s protocol.
